# An embryonic atrazine exposure results in reproductive dysfunction in adult zebrafish and morphological alterations in their offspring

**DOI:** 10.1038/srep21337

**Published:** 2016-02-19

**Authors:** Sara E. Wirbisky, Gregory J. Weber, Maria S. Sepúlveda, Tsang-Long Lin, Amber S. Jannasch, Jennifer L. Freeman

**Affiliations:** 1 School of Health Sciences, West Lafayette, IN, 47907, USA; 2 Department of Forestry and Natural Resources, West Lafayette, IN, 47907, USA; 3 Department of Comparative Pathobiology, West Lafayette, IN, 47907, USA; 4 Bindley Bioscience Center, Purdue University, West Lafayette, IN, 47907, USA.

## Abstract

The herbicide atrazine, a suspected endocrine disrupting chemical (EDC), frequently contaminates potable water supplies. Studies suggest alterations in the neuroendocrine system along the hypothalamus-pituitary-gonadal axis; however, most studies address either developmental, pubertal, or adulthood exposures, with few investigations regarding a developmental origins hypothesis. In this study, zebrafish were exposed to 0, 0.3, 3, or 30 parts per billion (ppb) atrazine through embryogenesis and then allowed to mature with no additional chemical exposure. Reproductive function, histopathology, hormone levels, offspring morphology, and the ovarian transcriptome were assessed. Embryonic atrazine exposure resulted in a significant increase in progesterone levels in the 3 and 30 ppb groups. A significant decrease in spawning and a significant increase in follicular atresia in the 30 ppb group were observed. In offspring, a decrease in the head length to body ratio in the 30 ppb group, along with a significant increase in head width to body ratio in the 0.3 and 3 ppb groups occurred. Transcriptomic alterations involved genes associated with endocrine system development and function, tissue development, and behavior. This study provides evidence to support atrazine as an EDC causing reproductive dysfunction and molecular alterations in adults exposed only during embryogenesis and morphological alterations in their offspring.

Studies investigating the effects of early life exposure to environmental stressors or stimuli have increased dramatically over the past decade. These studies seek to investigate the developmental origin of health and adult disease (DOHaD) hypothesis which states that exposure to stressors during sensitive times during an organism’s life, specifically during developmental stages, can cause changes to the genome and epigenome thereby resulting in an increased susceptibility to the development of health issues or diseases later on in life^1^,^2^. A key element complicating the establishment of a link between exposure and a disease state is the time that elapses between exposure and outward response or development of a disease^1^,^3^. Thus, it may take years for an individual to present a disease state and in addition may pass on these adverse health effects to future generations^4^.

Endocrine disrupting chemicals (EDCs) are exogenous agents that alter endocrine system functions and are associated with a myriad of diseases. In recent years, public concern about the effects of EDCs on human health has increased substantially and heightened the need for further research into the underlying molecular mechanisms of toxicity of these compounds^5^,^6^. EDCs are diverse in structure and are present in many products such as pharmaceuticals, plasticizers, and pesticides, making human exposure to these potentially harmful compounds a likely event. Evidence suggests that EDCs do not adhere to classic dose-response toxicological principles; rather they are part of the ‘low dose hypothesis’ due to their ability to disrupt hormonal homeostasis at low concentrations^7^. Studies show that EDCs can cause irreversible changes in tissue formation, decreased reproductive potential, obesity, and cancer^8^,^9^,^10^,^11^,^12^. Moreover, evidence suggests that exposure to EDCs can cause adverse effects not only in organisms that come into contact with them, but also to future progeny of exposed individuals^13^.

Atrazine (2-chloro-4-ethylamino-6-isopropylamino-1,3,5-triazine) is a pre-emergent herbicide predominately used in the Midwestern United States to control broadleaf and grassy weeds on a variety of field crops^14^. Exposure to atrazine can occur through different routes including ingestion of contaminated drinking water and in occupational settings via inhalation^15^,^16^,^17^. Furthermore, atrazine is often reported to exceed the Maximum Contaminant Level (MCL) of 3 parts per billion (ppb; μg/L) set by the U.S. Environmental Protection Agency (EPA) in potable water supplies^18^,^19^. As such the European Union banned the use of atrazine in 2003^20^,^21^. Epidemiological studies show several potential adverse health effects associated with maternal atrazine exposure including an increased risk of babies born small for their gestational age (SGA), intrauterine growth retardation (IUGR), and birth defects^22^,^23^,^24^.

Reproductive dysfunction caused by atrazine exposure through the hypothalamus-pituitary-gonadal (HPG) axis has been investigated in female rodent models. Developmental and peripubertal studies report a delay in sexual maturation and mammary gland development^25^. Adult studies report an inhibition of gonadotropin releasing hormone (GnRH) and a reduction in the pre-ovulatory surge of luteinizing hormone (LH), follicle stimulating hormone (FSH), and prolactin (PRL)^8^,^26^,^27^. Furthermore, atrazine has been reported to increase progesterone (P4) levels and is hypothesized to contribute to ovarian degeneration and decreased levels of LH and FSH; potentially leading to early reproductive senescence and dysfunction^28^,^29^.

When investigating developmental toxicant exposure and the developmental origins paradigm, the zebrafish provides a strong complementary vertebrate model. There are multiple strengths associated with utilizing the zebrafish including *ex utero* fertilization and embryonic development, rapid embryogenesis, and a relatively short life span. Paired with these biological strengths are the structural and functional homology of the zebrafish central nervous system (CNS) to humans and the conserved genetic, molecular, and endocrine pathways making the zebrafish a powerful model to assess the later-in-life alterations caused by an embryonic atrazine exposure^30^,^31^.

We previously reported that an embryonic atrazine exposure of 0.3, 3, or 30 ppb in zebrafish larvae resulted in immediate alterations to the transcriptome with gene ontology analysis showing enrichment for genes associated with reproductive system function and development, cell cycle regulation, and cancer^32^. In addition, our previous study examining the DOHaD paradigm showed that an embryonic atrazine exposure alters serotonin turnover and its metabolite 5-hydroxyindoleacetic acid (5-HIAA) in adult female zebrafish with brain transcriptomic profiles indicating enrichment of genes associated with nervous system development and function, behavior, and tissue development^33^.

In the current study, we aimed to address the later-in-life consequences of an embryonic atrazine exposure by assessing effects and function of the reproductive system, transcriptomic analysis of adult female ovarian tissue, and morphological alterations in the exposed generation’s offspring (Supplementary Material, Figure S1). Gonad tissue transcriptomic analysis was compared to the previously completed analysis of brain tissue^33^ for further assessment of alterations within the HPG axis.

## Results

### Assessment of an embryonic atrazine exposure on adult zebrafish reproductive function and offspring viability and morphology

**Figure 1:**
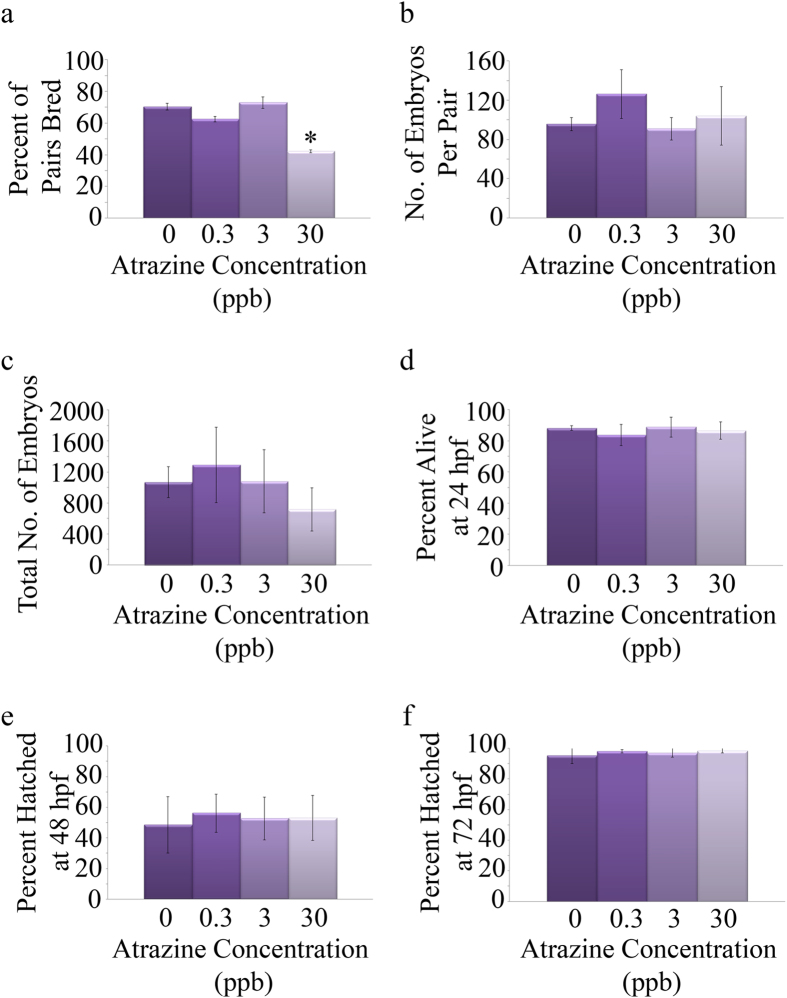
Assessment of embryonic atrazine exposure on adult zebrafish reproductive function and offspring viability. Adults were individually paired in mating experiments to assess mating success (16 pairs from each of the 4 biological replicates). Average number of pairs that bred was decreased in the group exposed to 30 ppb atrazine during embryogenesis (**a**). There were no significant differences observed for the number of embryos per pair or the total number of embryos per treatment (**b**,**c**, respectively). In addition, no significant changes were observed in mortality of the offspring (**d**) or in hatching rates at 48 and 72 hpf (**e**,**f**, respectively). Error bars are expressed as ± SD. (*p < 0.05).

**Figure 2:**
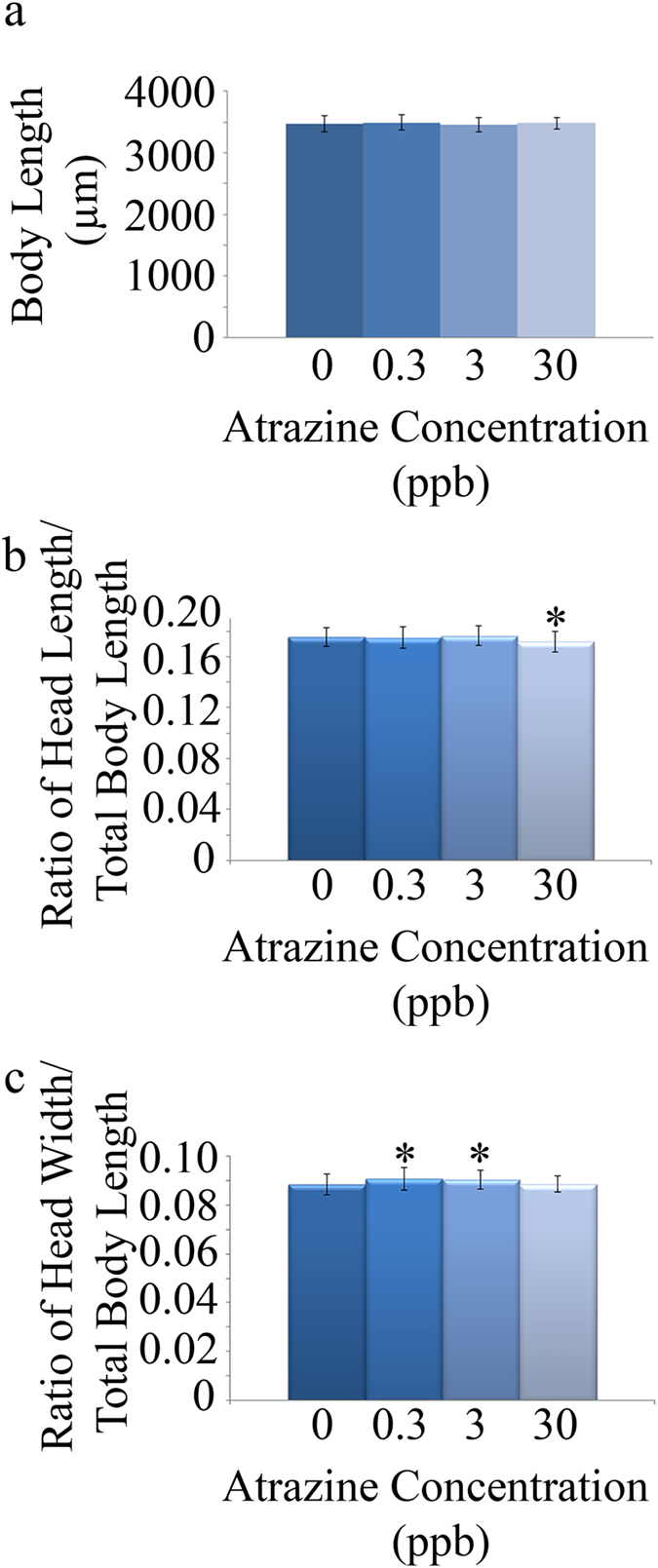
Morphological assessment of offspring. Twenty embryos (considered subsamples; n = 4) of the adult zebrafish population exposed to atrazine during embryogenesis were assessed at 72 hpf to obtain whole larvae total length (measured from snout to end of tail), head length, and head width. No significant alterations were found in total body length (**a**); however, a significant decrease in the head length to body length ratio in the 30 ppb atrazine treatment group was observed (**b**). In addition, a significant increase in the head width to body length ratio in the 0.3 and 3 ppb atrazine treatments was observed (**c**). Error bars are expressed as ± SD. (*p < 0.05).

We did not observe a skew in sex ratios in any of the treatment groups (p = 0.64; Supplementary Material, Figure S2). The average number of breeding pairs that spawned was significantly lower in the 30 ppb treatment group as compared to other treatment groups (p = 0.008; Fig. 1A), but the average number of embryos per pair (p = 0.21; Fig. 1B) and total number of live embryos in each treatment were not statistically different among treatments (p = 0.08; Fig. 1C). In addition, there were no statistically significant differences in mortality at 24, 48, or 72 hpf (at 24 hpf: p = 0.62; Fig. 1D; 48 and 72 hpf data not shown as there were no additional deaths) or hatching rates among the treatment groups (48 hpf: p = 0.64; Fig. 1E; 72 hpf: p = 0.43; Fig. 1F). Morphological characteristics of the offspring were also measured. While no significant alterations occurred in total body length (p = 0.43; Fig. 2A), a significant decrease in the ratio of head length to total body length in the 30 ppb breeding group (p = 0.0061; Fig. 2B) and a significant increase in the ratio of head width to total body length in the 0.3 and 3 ppb breeding groups were observed (p = 0.0011; Fig. 2C).

### Effects of an embryonic atrazine exposure on adult female zebrafish

**Figure 3:**
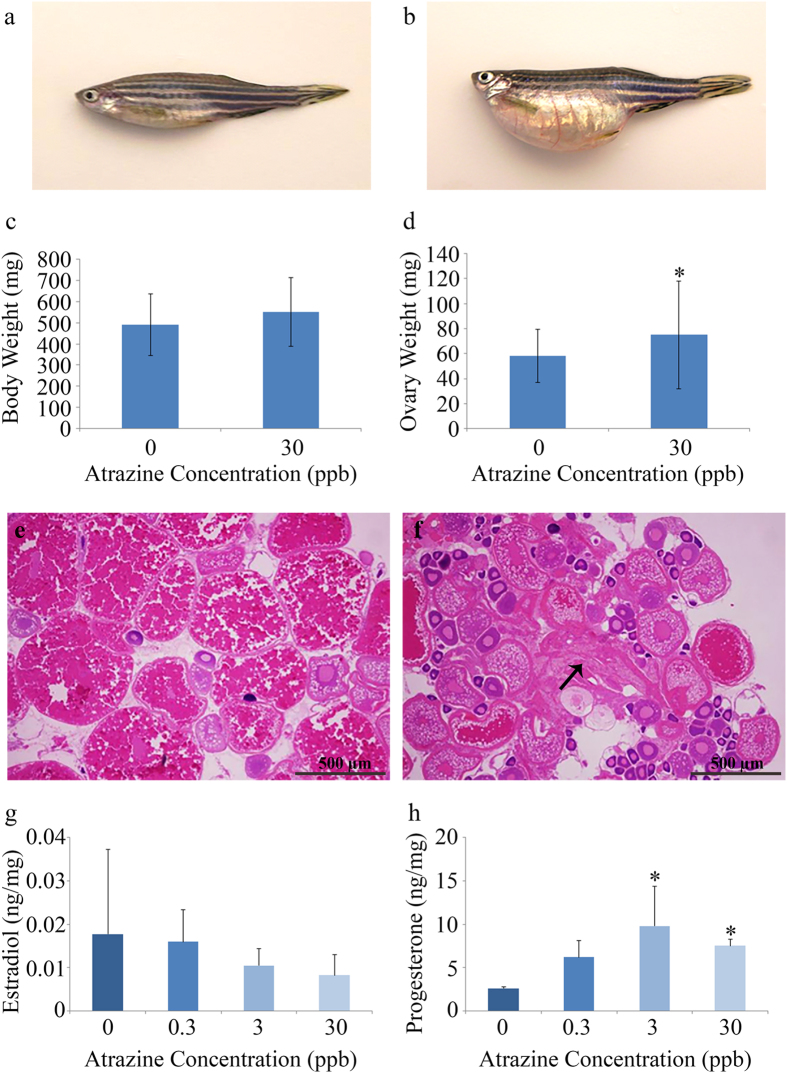
Effects of embryonic atrazine exposure on adult female zebrafish. A representative image of an adult female from the control group (**a**) compared to an adult female with a swollen abdomen in the 30 ppb developmentally exposed group (**b**). There was a ~5% incidence rate observed in this treatment group (~24–37 females assessed in each treatment of the four replicates). No significant difference in overall body weight was seen between the control and 30 ppb treatment group (**c**), but a significant increase in ovary weight was observed (**d**) (4 replicates with 10 female fish assessed per treatment replicate). Moreover, in comparison to the normal adult female zebrafish (**e**) an increase in atretic ovarian follicles (black arrow) was observed in those fish that presented with abdominal swelling (**f**) determined to be from an inability to release eggs (4 replicates with 10 female fish assessed per treatment replicate). Furthermore, while no significant differences were observed in estradiol (**g**), a significant increase in progesterone (P4) was present in adult females exposed to 3 or 30 ppb atrazine during embryogenesis (**h**) [4 to 7 adult females were pooled for each biological replicate (n = 4)]. Error bars are expressed as ± SD. (*p < 0.05).

Approximately 5% of the females from the 30 ppb treatment groups displayed an increase in abdominal swelling (Fig. 3A,B). Two of these individuals had severe swelling to the point of rupture. Acid-fast Ziehl-Neelsen staining revealed absence of mycobacterial organisms in these females indicating abdominal swelling was likely not due to infection (data not shown). Pathological assessment indicated swelling was due to the inability to release eggs. Several endpoints were then assessed to further investigate this observation. No significant differences were observed in the total weight of females in the 30 ppb treatment groups compared to the control treatment group (p = 0.09; Fig. 3C), but there was a significant increase in ovarian weight (p = 0.03; Fig. 3D). There was also no significant difference in GSI (p = 0.11; Supplementary Material, Figure S3). Ten individual females were then analyzed for differences in follicular staging in each of the different treatment groups in each of the four replicates (40 total female fish assessed). The percent follicles in different stages (perinuclear, cortical alveoli, early and late vitellogenic, and post-ovulatory) and the percent of atretic follicles did not differ across treatments (Supplementary Material, Figure S4A), but when specifically evaluating females exhibiting swollen abdomens in comparison to those that were not, a significant increase in the number of atretic follicles was observed (p = 0.0002; Fig. 3E,F; Supplementary Material, Figure S4B).

### Estradiol and progesterone levels in adult female ovarian tissue

No significant alterations were observed in ovarian tissue concentrations of estradiol in any of the atrazine treatments (p = 0.5337; Fig. 3G). However, a significant increase in progesterone concentrations were observed in the 3 and 30 ppb atrazine treatment groups (p = 0.0043; Fig. 3H).

### Transcriptome analysis of adult female gonad tissue

Transcriptomic profiling of ovaries was assessed to investigate the genetic mechanisms underlying alterations in reproductive success. Results showed expression alterations in 2,024 mapped genes in the 0.3 ppb treatment group, 843 genes in the 3 ppb treatment group, and 696 genes in the 30 ppb treatment group (GSE73740). Of these differentially expressed genes, 383 were common among all three atrazine treatments (Supplementary Material, Table S1). Gene ontology analysis revealed embryonic development, behavior, and organismal survival were enriched for the 0.3 ppb treatment group (Supplementary Material, Table S2). The 3 ppb treatment group showed a similar response as pathways enriched showed changes to genes involved in organ morphology, behavior, and organismal survival (See Supplementary Material, Table S3). Pathways enriched in the 30 ppb treatment group included tissue development, behavior, and organismal survival (See Supplementary Material, Table S4). Gene ontology analysis of the 383 genes altered in all three treatment groups revealed effects in endocrine system development and function, tissue development, and behavior (Table 1).

### qPCR confirmation of microarray data

A subset of genes detected to be significantly altered by microarrays in all atrazine treatment groups was independently confirmed by qPCR with all seven target genes (*STAR, ACE, CYP19A1, CYP1B1, CRHBP, HPGD*, and *VIP*) significantly changed in the qPCR assessment (Table 2).

## Discussion

In order to assess how an embryonic atrazine exposure affects adult reproductive system function we performed paired breeding experiments to assess mating success, spawning, and survival of the progeny. We observed a significant decrease in the number of females that spawned in the 30 ppb treatment group. Several females in the 30 ppb treatment group presented with swollen abdomens due to an inability to spawn supporting the lack of successful mating with an increase in atretic ovarian follicles present in these individuals. Previous studies have shown that atrazine exposure causes a decrease in spawning events^34^,^35^. Tillitt *et al.*^34^ exposed adult fathead minnows to 0.5, 5, or 50 ppb atrazine for 14 or 30 days resulting in a decrease in spawning events and cumulative mean egg production with increasing atrazine concentrations^34^. A second study conducted by the same group exposed adult Japanese medaka to similar atrazine concentrations and confirmed the decrease in mean egg production; however, a decrease in spawning events was not observed^35^. Interestingly, we observed a similar effect in our current study in adult zebrafish exposed to atrazine only during embryogenesis. Reductions in spawning could be attributed to either a male or female specific effect on reproductive behavior. Although direct breeding behavior was not assessed in our study, previous studies have noted alterations in reproductive behavior in various fish species with atrazine exposure^36^,^37^.

Offspring from the successful matings were collected and grown to the end of the embryonic phase to observe mortality, hatching rates, and morphology. While no significant differences were observed for mortality or hatching rate, a decrease in head length-to-body ratio in offspring from the 30 ppb treatment group and an increase in head width-to-body ratio in offspring from the 0.3 and 3 ppb treatment groups was observed. A previous study from our laboratory reported an increase in head length and head-to-body ratio in zebrafish larvae exposed to 0.3, 3, or 30 ppb atrazine through embryogenesis^32^. In addition, epidemiological studies report a link between *in utero* atrazine exposure and an increased risk for babies born small for their gestational age (SGA) as well as impairments to fetal growth and birth defects^22^,^24^,^38^. While these studies assess the immediate effects of atrazine exposure during development, the results of the current study indicate that an embryonic atrazine exposure can also stimulate and repress the development of offspring of the exposed generation.

Due to our observed abdominal swelling and decrease in spawning events in the 30 ppb treatment group, gonadal tissue was collected from adult females in each of the treatment groups and subsequent histological analysis of ovaries was performed to observe frequency of different oocyte stages. Although we found no significant differences in oocyte stage across the treatment groups, an increase in atretic ovarian follicles was observed in females with abdominal swelling. Moreover, the fish with this phenotype tested negative for mycobacteria, which is also reported to elicit swollen abdomens in zebrafish. It should be noted that we did observe females with larger abdomens in the other atrazine treatment groups, albeit not to the extent that was observed in the 30 ppb treatment group. In addition, there were no females that presented with this phenotype in the control groups and we have never observed a similar effect in our breeding groups. A previous study exposed female Sprague-Dawley rats to 3, 30, or 300 mg/kg atrazine for two or four weeks reported a prolonged diestrus period, large-sized atretic follicles, and uterine atrophy in the 300 mg/kg treatment group^39^. Furthermore, a study conducted by Gojmerac *et al.*^28^ reported that an atrazine exposure of 2 mg/kg in feed for 19 days in adult female pigs caused cystic ovarian degeneration and an increase in the persistence of the corpus luteum^28^. Reproductive hormones play a key role in maintaining reproductive homeostasis. Of primary importance is LH which plays a key role in follicular development and normal reproductive function^26^,^40^. Studies show that atrazine reduces the release of LH from the pituitary gland^26^,^41^. Therefore, the results observed here and in previously mentioned studies suggest an anovulatory effect of atrazine which could be due to the previously observed reduction in LH, providing a mechanism behind the observed reduction in spawning, atretic follicles, and cystic ovarian degeneration^39^.

In addition, this study is innovative in that we are the first to show an increase in ovarian progesterone (P4) in adult female zebrafish exposed to 3 or 30 ppb atrazine only during embryogenesis. A study conducted by Gojmerac *et al.*^28^ reported that an atrazine exposure of 2 mg/kg in feed for 19 days in adult female pigs caused a significant increase in P4^28^. As previously discussed, results from this study also included an increase in cystic ovarian degeneration. It was shown that higher levels of P4 inhibit follicle development and can potentiate follicular atresia^29^. Additional *in vivo* studies report that atrazine exposure in Long-Evans Hooded and Sprague Dawley rats also caused significant increases in P4^42^,^43^,^44^. As previously noted, atrazine exposure decreased LH and FSH leading to reproductive dysfunction^8^,^26^,^45^. Furthermore, increases in P4 may contribute to the inhibition of the pre-ovulatory surge of LH from the pituitary enhancing reproductive dysfunction. Furthermore, *in vitro* studies utilizing swine and rat granulosa cells have reported increases in P4 following atrazine exposure^46^,^47^. An *in vitro* study conducted by Pogrmic-Majkic *et al.*^47^ provided mechanistic data regarding the observed increase in P4 elicited by atrazine exposure. Results suggest that atrazine causes an increase in P4 and overexpression of luteal markers (*STAR* and *CYP11A1*) through the stimulation of the cAMP, AKT, and CEBPB-signaling pathways^47^. The rise in P4 (a critical step in early luteinization) was also demonstrated in H295R adrenal cortical carcinoma cells^48^. The findings that atrazine elevates P4 combined with the observed follicular atresia indicates the ability of atrazine to alter ovarian function and reduce fertility following only a developmental exposure. Results from our study also revealed no significant alterations in ovarian levels of 17β-estradiol. Current literature surrounding the effects of atrazine on estrogen is primarily conducted in male animal models; while levels of LH, FSH, PRL, P4, and gonadotropin releasing hormone (GnRH) were the forefront in female models^8^,^27^,^44^,^45^.

Transcriptomic analysis was also completed on the ovarian tissue of adult female zebrafish exposed to atrazine during embryogenesis in order to identify genetic alterations behind the observed decrease in spawning and increase in P4. Analysis revealed a subset of genes altered that are involved in endocrine system development and function. The first key gene of interest is steroidogenic acute regulatory protein (*STAR*). This protein is responsible for the transport of cholesterol from the outer to inner mitochondrial membrane where it is then metabolized to pregnenolone^49^. A study conducted by Pogrmic *et al.*^49^ showed a decrease in *STAR* and other steroidogenic genes (scavenger receptor B1 (*SR-B1*), steroidogenic factor 1 (*SF-1*), cytochrome P450 17A1 (*CYP17A1*), and 17β-hydroxysteroid dehydrogenase (*17β-HSD*)) in Leydig cells after exposure from PND 23–51 to 50 or 200 mg/kg/day atrazine^49^. A second study conducted by Pogrmic-Majkic *et al.*^50^ only treated Leydig cells for 24 hours with 20 μM atrazine which caused an increase in expression of *STAR*, *SF-1*, and *17β-HSD*^50^. The results from these studies led to the hypothesis of a potential transient stimulatory effect of atrazine. This up regulation of *STAR* was also observed in FSH stimulated granulosa cells after atrazine exposure^47^. Results from these previous studies indicate that atrazine can elicit alterations in *STAR*. Our results provide new insight into the long-term effects of atrazine on female reproductive function due to the increase in *STAR* expression.

One of the most controversial genes associated with atrazine exposure is aromatase (*CYP19A1*). Aromatase is responsible for catalyzing the conversion of testosterone to estradiol as well as androstenedione to estrone in the steroidogenic pathway^51^. Transcriptomic results from our study reveal an increase in *CYP19A1* expression in all three atrazine treatments approximately six months after exposure completion. Although an increase in *CYP19A1* was observed in ovarian tissue, no alterations were observed in levels of gonadal estradiol as is hypothesized^52^,^53^. However, further investigation is warranted to determine if the increase in *CYP19A1* elicits a response in plasma levels of estradiol and a corresponding decrease in testosterone. In 2002 and 2010, Hayes *et al.* reported that atrazine exposure resulted in hermaphroditic, demasculinized, and chemically castrated *X. laevis* at concentrations as low as 0.1 and 2.5 ppb (μg/L)^52^,^53^. Hayes *et al.*^53^ hypothesized that the observed morphological alterations were due to an increase in *CYP19A1* expression which would therefore lead to an increase in estrogen and a decrease in testosterone^53^. This hypothesis has been under investigation as other studies have not demonstrated that atrazine causes increases in *CYP19A1* expression in *X. laevis* and other aquatic species^54^,^55^,^56^,^57^,^58^. *In vitro* studies report contrasting results indicating that atrazine exposure alters aromatase mRNA and activity in human cancer cell lines^48^,^59^,^60^,^61^ and luteinized granulosa cells^51^.

An additional gene altered in ovarian tissue was the neuropeptide adenylate cyclase-activating peptide 1 (*ADCYAP1*) which regulates gonadotropin gene expression. *ADCYAP1* has widespread distribution and function which includes expression in the central and peripheral nervous system, adrenal glands, placenta, ovaries, and testes^62^. Estradiol and P4 stimulate the expression of *ADCYAP1* in the hypothalamus and ovary^63^,^64^,^65^,^66^. The observed increase in *ADCYAP1* expression in ovary tissue corresponds to the observed increase in P4 levels. Additionally, our previous study examining the effects of a developmental atrazine exposure on female brain tissue also revealed an alteration in *ADCY* upon transcriptomic analysis^33^ as it works synergistically with GnRH, therefore playing a role in the release of LH and FSH^62^. All three are needed for proper reproductive function and each is shown to be decreased by atrazine exposure^8^,^27^,^40^. Furthermore, as previously stated, atrazine increases P4 through the stimulation of the cAMP, AKT, and CEBPB-signaling pathways^47^. CREB signaling proteins are also identified as a stimulator of *ADCYAP1*^67^.

An increase was also observed in *CYP1B1* in all three atrazine treatments. *CYP1B1* is expressed constitutively in steroidogenic tissues such as the adrenal, ovary, and testes^68^. Therefore, its regulation is primarily by hormones that elevate cAMP. Steroidogenic cytochrome P450’s undergo transcriptional regulation by numerous transcription factors such as *SF-1*. *SF-1* is a transcriptional regulator for *CYP19A1*, *STAR*, and *CYP11A1*. Although this gene is heavily integrated within the steroidogenic pathway, its up regulation may provide further insight into the mechanisms behind the adverse effects of atrazine exposure, especially those effects lasting into adulthood.

An additional gene altered by all three atrazine treatments was vasoactive intestinal peptide (*VIP*). *VIP* mRNA is found in the ovaries of rodents, specifically, in the granulosa layer of the pre-ovulatory follicles. *VIP* acts on specific targets which activates adenylyl cyclase pathway, therefore stimulating the cAMP production. This gene stimulates progesterone, androgens, and estradiol secretion. It is also associated with the initiation of follicular growth and the inhibition of follicular atresia^69^. *VIP* was down regulated in our transcriptomic analysis; this down regulation could play a role in the observed follicular atresia observed. The observed upregulation of *ADCYAP1* may be a compensatory mechanism to the down regulation of *VIP* to continue the release of steroid hormones.

Additionally, it is known that EDCs can affect many aspects of the hormonal pathway including their synthesis, transport, excretion, and metabolism^7^. Our microarray and qPCR analysis show that embryonic atrazine exposure elicits an up-regulation of the *CRHBP* gene. This gene is a potent stimulator of the synthesis and secretion of preopiomelanocortin-derived peptides such as α-Melanocyte-stimulating hormone (α-MSH) and adrenocorticotropic hormone (ACTH). Although these additional hormones are highly associated with the body’s primary stress pathway (hypothalamus-pituitary-adrenal (HPA) axis); this axis works in tandem with the HPG axis. Gene ontology revealed *CRHBP* to aid in the cellular response to GnRH. GnRH is a key regulator of the HPG axis and has been the focus of studies aiming to elucidate the mechanism of atrazine toxicity^27^,^40^.

**Figure 4:**
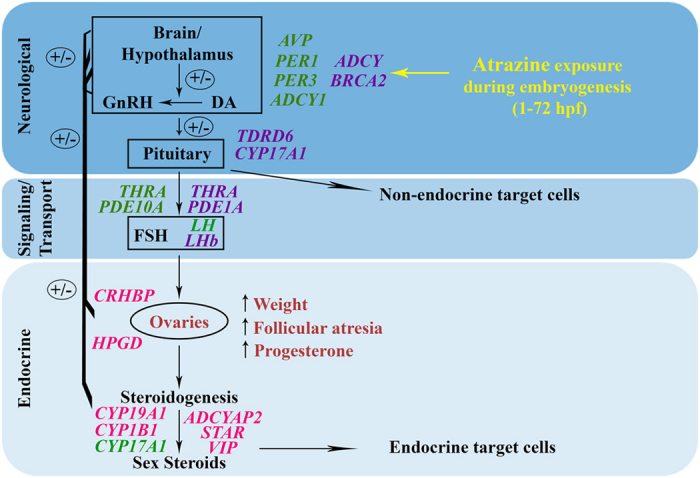
Schematic diagram of the effects of an embryonic atrazine exposure on the HPG axis in female zebrafish. Diagram representing the effects of an embryonic atrazine exposure at 72 hpf as well as in brain and ovarian tissue of adult females exposed during embryogenesis compiling the data from this study, Weber *et al.*^32^, and Wirbisky *et al.*^33^. The genes in green are representative of changes observed at 72 hpf; genes in purple are altered in adult female brain tissue; genes listed in pink are those altered in adult female gonad tissue; and red signifies the morphological alterations observed.

Our data suggests a developmental origin of reproductive dysfunction in adult female zebrafish caused by an embryonic atrazine exposure. Reproductive traits, morphological and hormone data, and gene expression analysis provide a conceptual working model of atrazine toxicity when coupled to our previous studies^32^,^33^ (Fig. 4). Indeed, many adult diseases and disorders are believed to originate from exposure to environmental stressors during development by altering and reprogramming normal cellular processes that lead to an altered physiological state. The disruption of the reproductive system through the developing neuroendocrine system was identified in our initial gene expression assessment^32^. From our transcriptomic analysis, we reported a disruption in the expression of *CYP17A1*, *LH*, and *ADCY1*. These genes are critical for steroidogenesis and proper reproductive function. Additionally, alterations in *PER1* and *PER3* were observed which play a role in regulating circadian rhythm; a process vital for maintaining homeostatic reproductive function^70^. We also reported alterations in *THRA*; a gene responsible for proper thyroid function at 72 hpf and in adult female brain tissue following the developmental atrazine exposure^32^,^33^. Although the primary effect of atrazine exposure is reported throughout the HPG axis, results from our developmental and adult studies show that the hypothalamus-pituitary-thyroid (HPT) axis may also be disrupted, although further studies are necessary. In addition, we observed alterations in *PDE10A* following embryonic atrazine exposure as well as *PDE1A* in adult brain tissue. Phosphodiesterase genes are reported to be affected by atrazine exposure^71^,^72^. Alterations in these upstream cellular regulators can have an adverse effect on steroidogenesis through cAMP. Transcriptomic profiling of the brain tissue of females exposed to atrazine only during embryogenesis also revealed enrichment in genes associated with neurological system development and function, organ and tissue development, body size, and behavior as well as a decrease in the serotonin metabolite 5-HIAA and serotonin turnover^33^. Although neurotransmitter systems were not addressed in the results shown here, neurotransmitters and neuropeptides that are involved in GnRH release are reported to be altered by atrazine exposure^73^,^74^,^75^. Furthermore, transcriptomic profiling of ovarian tissue of females exposed to atrazine during embryogenesis revealed enrichment of genes associated with endocrine system development and function and steroidogenesis (*CYP19A1*, *CYP1B1*, *ADCYAP2*, *STAR*, *VIP*) providing a genetic link to the observed reproductive dysfunction, follicular atresia, and increased levels of progesterone. The results from these and previous studies provide support for atrazine exposure causing adverse effects to the neuroendocrine system immediately following atrazine exposure as well as later in life.

## Methods

### Zebrafish husbandry and experimental design

Zebrafish (wild-type AB strain) were housed in a Z-Mod System (Aquatic Habitats) on a 14:10 hour light:dark cycle and maintained at 28 °C with a pH of 6.9–7.2 and conductivity range of 470–520 μS. Adult zebrafish were bred in cages and embryos were collected, staged, and rinsed with system water as described previously for experimental use^76^. Embryos were dosed with 0, 0.3, 3, or 30 ppb atrazine (CAS #1912-24-9; Chem Service, 98% purity) from 1–72 hours post fertilization (hpf) as previously described^32^,^33^. Atrazine sample concentrations were verified using an U.S. EPA approved immunoassay kit for atrazine (Abraxis Atrazine ELISA kit, Warminster, PA) as previously described^77^. After exposure, larvae were rinsed with clean fish system water, housed in 4-liter tanks in the system with 20–30 fish per tank, and allowed to mature under normal growing conditions (Supplementary Material, Figure S1). Biological replicates are defined by groups started on different days. All animal protocols were approved and performed in accordance with Purdue University’s Institutional Animal Care and Use Committee guidelines.

### Assessment of reproductive potential and morphology of offspring

At approximately 5 months post fertilization (mpf) zebrafish from each treatment group (0, 0.3, 3, or 30 ppb atrazine) were sexed and bred within respective treatment groups in 4-liter tanks once a week for three continuous weeks in order to initiate breeding cycles (Supplementary Material, Figure S1). To assess individual breeding potential, one male and one female from each treatment group were randomly selected and paired for breeding. A total of 64 individual pairs were tested for each treatment group among the four biological replicates (16 pairs per replicate). The number of pairs that spawned was noted and embryos from each pair were collected and observed through embryogenesis (72 hpf) noting: the number of fertilized embryos and unfertilized eggs per pair; mortality at 24, 48 and 72 hpf; and number of embryos hatched at 48 and 72 hpf. In addition, twenty larvae from each treatment dish (considered subsamples) of each biological replicate (n = 4) were analyzed with light microscopy using a Nikon SMZ1500 dissecting scope with NIS Elements imaging software to attain total body length (measured from snout to end of tail), head length, and head width.

### Fish gonadal tissue collection and histology

Adult female zebrafish (approximately 5–8 mpf) were euthanized in MS-222 (Ethyl 3-aminobenzoate methanesulfonate, Sigma, St. Louis, MO) (4 mg/mL) and subsequently weighed to obtain body weight (4 replicates with 10 fish per replicate). Ovaries were collected and weighed for calculation of gonadal somatic index (GSI) determined as (gonad weight/total tissue weight)*100. A sample of ovarian tissue was collected from both ovaries and fixed in Davidson’s Fixative^78^ overnight at room temperature and then transferred to histology grade 70% EtOH (Sigma). Hematoxylin and eosin (H & E) sections were prepared following standard procedures and sections examined under a light microscope (10–40X) for staging and evaluation of any abnormalities as outlined in Johnson *et al.*^78^. All follicles were counted and classified as perinuclear, cortical alveoli, early vitellogenic, late vitellogenic, post-ovulatory, or atretic. Frequencies of occurrence of each stage were then calculated from each section and averaged.

### Estradiol and progesterone analysis in adult female ovary tissue

Ovarian tissue from adult females (~6 mpf) was collected from each treatment group. Four to seven adult females were pooled for each biological replicate (n = 4). Tissue was stored at −80 °C prior to extraction and analysis. Samples were weighed and 100 mg of tissue placed in a 1.7 mL tube. 1000 ng of d_5_-estradiol or 100 ng of d_9_-progesterone was added as an internal standard to each tube. Glass homogenizer beads and water were added, tubes briefly vortexed, and then 1 mL of hexane/ethyl acetate (60/40 v/v) added to each. The samples were vortexed for 15 minutes on maximum speed and then centrifuged at 15,000 rpm for 5 minutes. The top organic layer was transferred to a new tube, dried in a rotary evaporation device, and stored at −20 °C until derivatization.

For estradiol analysis the extracts were derivatized with dansyl chloride just prior to LC/MS/MS analysis^79^. Acetonitrile, 10mM sodium carbonate, and freshly prepared dansyl chloride was added to each sample for a reaction time of 10 minutes at 60 °C. For progesterone analysis the extracts were derivatized with the AB Sciex Keto derivatization kit (AB Sciex, Framingham, MA) just prior to LC/MS/MS analysis. 50 μL of reagent was added to each sample with a reaction time of 60 minutes at room temperature. The samples were transferred to autosampler vials and immediately analyzed on the instrument.

An Agilent 1200 Rapid Resolution liquid chromatography (LC) system coupled to an Agilent 6460 series QQQ mass spectrometer (MS) was used to analyze estradiol in each sample. A Waters Xbridge C18 2.1 mm × 100 mm, 3 μm column was used for LC separation. The buffers were (A) water + 0.1% formic acid and (B) acetonitrile + 0.1% formic acid. The linear LC gradient was as follows: time 0 minutes, 10% B; time 1 minute, 10% B; time 5 minutes, 100% B; time 15 minutes, 100% B; time 15.5 minutes, 10% B; time 18 minutes, 10% B. The flow rate was 0.3 mL/min. Multiple reaction monitoring was used for MS analysis. The data were acquired in positive electrospray ionization (ESI) mode by monitoring the following transitions: Estradiol-dansyl chloride 506.1→155.8 (40V), 171 (30V); d5-Estradiol-dansyl chloride 511.1→155.8 (40V), 171 (30V); Progesterone 429.1→370 (20V), 126 (30V); d_9_-Progesterone 438.1→379 (20V), 132 (30V). The jet stream ESI interface had a gas temperature of 325 °C, gas flow rate of 8 L/minute, nebulizer pressure of 40 psi, sheath gas temperature of 250 °C, sheath gas flow rate of 7 L/minute, capillary voltage of 4000 V, and nozzle voltage of 1000 V.

### Transcriptome microarray analysis of adult female gonad tissue

Ovarian tissue from six adult females (approximately 5–8 mpf) was collected among the groups exposed to an atrazine treatment (0, 0.3, 3, or 30 ppb) during embryogenesis, homogenized in Trizol (Life Technologies), and flash frozen in liquid nitrogen (n = 6). Total RNA was isolated by the RNeasy Mini Kit (Qiagen). Transcriptomic microarray analysis was conducted using the one-color hybridization strategy to compare gene expression profiles among the atrazine treatments with a custom zebrafish 4 × 180K expression platform (Agilent Technologies). This microarray is a multiplex format of 4 arrays each consisting of 180,000 probes interrogating 36,000 known and predicted targets with approximately 3–5 probes per target and is based on the Ensembl and UCSC Genome Databases. Following hybridization, arrays were washed and then scanned on an Agilent Technologies SureScan Microarray Scanner (Agilent Technologies). Array image data was extracted using Agilent Feature Extraction Software 11.5 (Agilent Technologies,). Data was uploaded to GeneSpring 12.5 (Agilent Technologies) for statistical analysis. Microarray analysis was performed following MIAME guidelines^80^. Each gene list was imported into Ingenuity Pathway Analysis (IPA) for gene ontology and molecular pathway analysis. Genes referred to in the results and discussion sections are reported as the human homologs of the genes identified to be altered by microarrays.

### Quantitative polymerase chain reaction (qPCR) confirmation of microarray

qPCR was performed on a subset of selected genes altered in the gene expression array analysis: *STAR, ACE, CYP19A1, CYP1B1, CRHBP, HPGD*, and *VIP* using the BioRad SSOAdvance SYBR Green Supermix kit according to the manufacturer’s recommendations. Probes specific to target genes were designed using the Primer3 website (Supplementary Material, Table S5). qPCR was performed following similar methods as previously described^32^,^33^ following the minimum information for publication of quantitative real-time PCR experiments (MIQE) guidelines^81^. Similar to as performed in previous studies in our laboratory several genes were assessed to determine the best reference gene to be used for this data set (data not shown)^32^,^33^. *β-ACTIN* was found to be the most consistent and least variable for this analysis. qPCR was performed on the same samples as used in the microarray analysis (n = 6). Experimental samples were run in triplicate (technical replicates) and gene expression was normalized to *β-ACTIN*. Efficiency and specificity were checked with melting and dilution curve analysis and no-template controls.

### Statistical analysis

The average number of embryos per treatment group, number of embryos per pair, survival, hatching success, spawning rates, sex ratios, morphology measurements of offspring, and gene expression array confirmation by qPCR were analyzed using an Analysis of Variance (ANOVA) with a least significant difference (LSD) post-hoc test when a significant ANOVA was observed (α = 0.05) using SAS statistical software. Total body weight and ovary weights between the control and 30 ppb treatment groups was analyzed with a T-test (p < 0.05). Counts of ovarian follicles and corresponding stages of development were assessed using a combination of Chi-square and a generalized linear mixed model (p < 0.05) in SAS statistical software. Hormone analysis was performed using an ANOVA and Tukey’s post-hoc test when a significant ANOVA was observed (α = 0.05). Microarray analysis was completed with an ANOVA and a Tukey’s post-hoc test when a significant ANOVA was observed (α = 0.05). In addition, a mean absolute log_2_ expression ratio of at least 0.585 (50% increase or decrease in expression) must be satisfied.

## Additional Information

**How to cite this article**: Wirbisky, S. E. *et al.* An embryonic atrazine exposure results in reproductive dysfunction in adult zebrafish and morphological alterations in their offspring. *Sci. Rep.* **6**, 21337; doi: 10.1038/srep21337 (2016).

**Accession Codes**: Transcriptomic data discussed in this publication has been deposited in NCBI’s Gene Expression Omnibus and is accessible through GEO Series accession number GSE73740. (http://www.ncbi.nlm. nih.gov/geo/query/acc.cgi?acc=GSE73740)

## Supplementary Material

Supplementary Information

## Figures and Tables

**Table 1:** Genes altered in all three atrazine treatments.

Physiological System Development and Function	p-value^a^	Number of Genes^b^
NERVOUS SYSTEM DEVELOPMENT AND FUNCTION	7.96E-06 – 1.85E-02	106
Growth of neurites	7.96E-06	27
Morphology of CNS	3.35E-05	28
Synaptic Transmission	1.16E-03	17
Differentiation of neurons	3.36E-04	20
ENDOCRINE SYSTEM DEVELOPMENT AND FUNCTION	1.52E-06 – 1.61E-02	22
Hormone Metabolism	6.75E-04	12
Steroidogenesis	6.40E-04	9
Accumulation of progesterone	6.76E-03	2
Concentration of corticosterone	1.23E-05	11
Synthesis of estrogen	6.26E-03	4
BEHAVIOR	2.85E-07 – 1.85E - 02	59
Behavior	2.85E-07	48
Cognition	2.91E-05	23
Learning	7.43E-04	19
Locomotion	7.91E-03	14
TISSUE DEVELOPMENT	7.96E-06 – 1.85E-02	78
Growth of neurites	7.96E-06	27
Gonadogenesis	4.99E-03	20
Development of genital organ	5.51E-03	21
Development of ovary	6.94E-03	8

^a^Derived from the likelihood of observing the degree of enrichment in a gene set of a given size by chance alone.

^b^Classified as being differentially expressed that relate to the specified function category; a gene may be present in more than one category.

**Table 2:** qPCR array confirmation of adult female gonad tissue.

Gene	SEQ_ID	ANOVA p-value (p < 0.05)	Up/Down
*STAR*	NM_131663.1	0.0134	Up
*ACE*	XM_005169447.1	0.019	Up
*CYP19A1*	NM_131154.2	0.0312	Up
*CYP1B1*	NM_001045256.1	0.0015	Up
*CRHBP*	XM_689239.5	0.0023	Up
*HPGD*	NM_001045256.1	0.0027	Up
*VIP*	NM_001114553.2	0.009	Down
