# Supplementary material for: An embryonic atrazine exposure results in reproductive dysfunction in adult zebrafish and morphological alterations in their offspring
Source: Sci Rep. 2016 Feb 19;6:21337. doi: 10.1038/srep21337 (PMC4759560; doi:10.1038/srep21337)
Supplement: Supplementary Information [file srep21337-s1.pdf]

Supplementary Material

An embryonic atrazine exposure results in reproductive dysfunction in adult zebrafish and morphological alterations in their offspring

Sara E. Wirbisky*, Gregory J. Weber*, Maria S. Sepulveda*†, Tsang-Long Lin[ϒ], Amber S. Jannasch[ǂ], Jennifer L. Freeman*[1]

*School of Health Sciences, †Department of Forestry and Natural Resources, [ϒ]Department of Comparative Pathobiology and [ǂ]Bindley Bioscience Center, Purdue University, West Lafayette, IN, 47907

Table of Contents:

I. Supplementary Material, Figure S1. Summary of exposure regimen and experimental analysis.
II. Supplementary Material, Figure S2. Sex ratios of adults exposed to atrazine during embryogenesis.
III. Supplementary Material, Figure S3. Average GSI of adult females among treatment groups.
IV. Supplementary Material, Figure S4. Follicular staging of adult female ovaries.
V. Supplementary Material, Table S1. Genes altered in adult female gonad tissue in all three atrazine treatments.
VI. Supplementary Material, Table S2. Gene enrichment table in adult female gonad tissue at 0.3 ppb atrazine.
VII. Supplementary Material, Table S3. Gene enrichment table in adult female gonad tissue at 3 ppb atrazine.
VIII. Supplementary Material, Table S4. Gene enrichment table in adult female gonad tissue at 30 ppb atrazine.
IX. Supplementary Material, Table S5. Primers used in qPCR confirmation of microarray analysis of female gonad tissue.

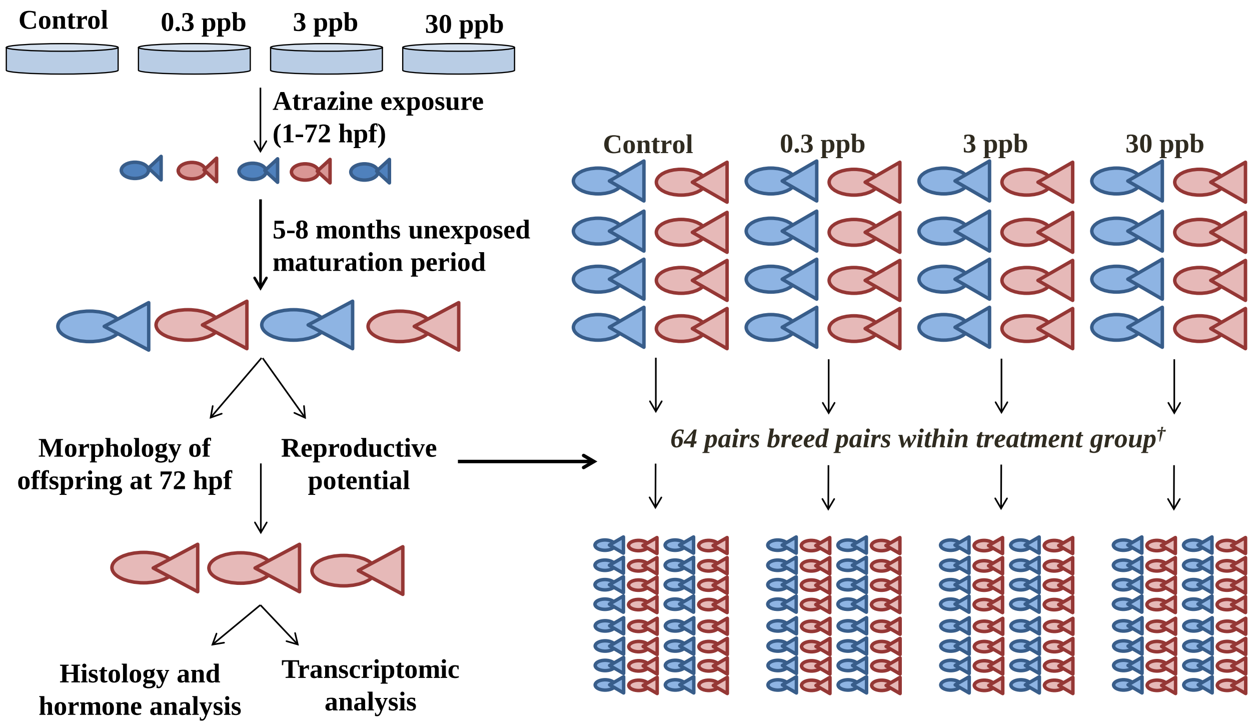

Supplementary Material, Figure S1. Summary of exposure regimen and experimental analysis. Zebrafish were bred and embryos were collected and treated with 0, 0.3, 3, or 30 ppb atrazine from 1-72 hpf. Following the exposure period, embryos were rinsed and allowed to mature under normal conditions until 5-8 mpf. Zebrafish were then bred for three weeks in order to initiate breeding cycles. To assess reproductive potential, single pairs were bred and number of pairs that bred, number of embryos per pair, total number of embryos per treatment, mortality, and hatching rates were assessed. In addition, offspring were collected for morphological assessment at 72 hpf. Adult female zebrafish were then collected for ovarian histology, hormone, and transcriptome analysis. (†16 pairs per replicate; 4 replicates; blue: male fish; pink: female fish)

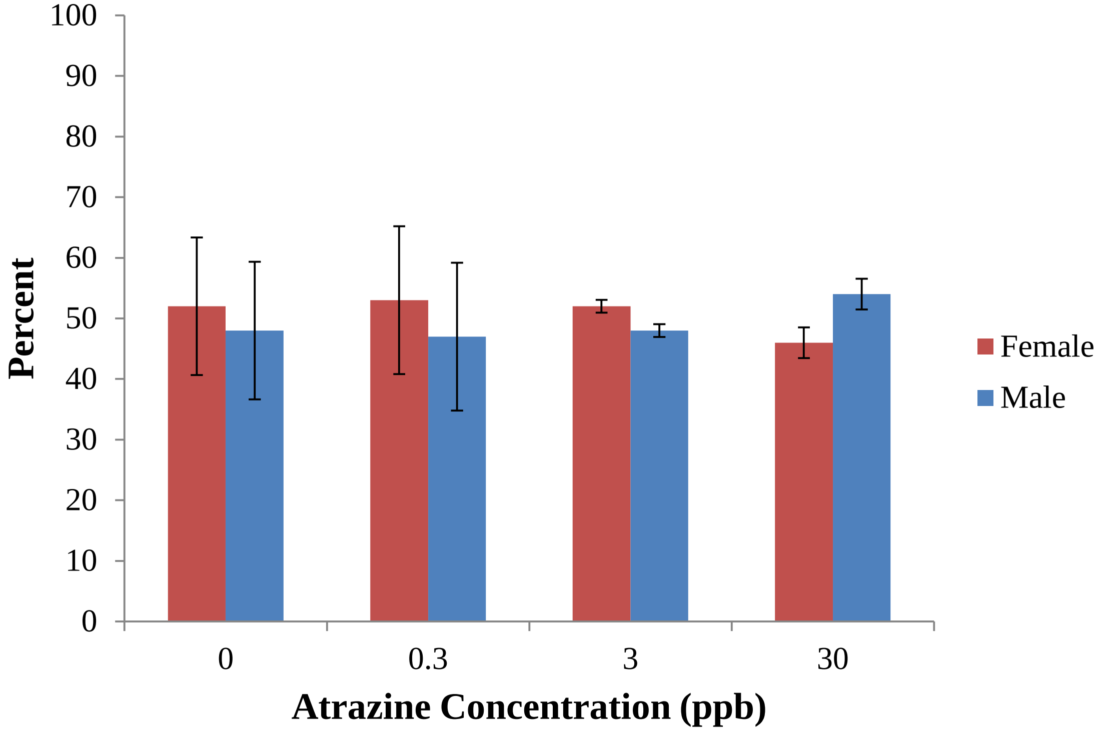

Supplementary Material, Figure S2. Sex ratios of adults exposed to atrazine during embryogenesis. Zebrafish were bred and embryos were collected and treated with 0, 0.3, 3, or 30 ppb atrazine from 1-72 hpf. Following the exposure period, embryos were rinsed and allowed to mature under normal conditions until 5-8 mpf. Zebrafish were then separated and counted based upon sex in order to determine sex ratios. No significant differences were observed among the treatment groups for sex ratios ($p=0.64$; 4 replicates with 60-75 fish per replicate treatment). Error bars are expressed as ± SD.

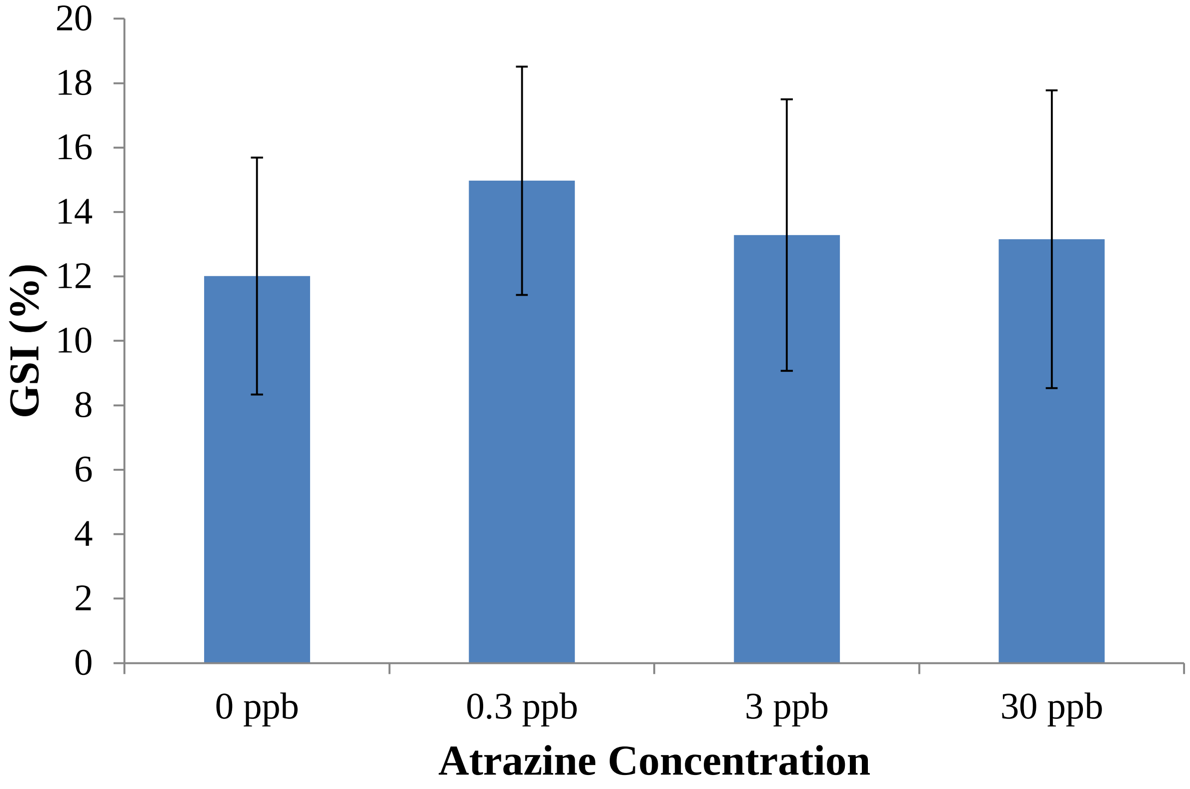

Supplementary Material, Figure S3. Average GSI of adult females among treatment groups. Adult female zebrafish (4 replicates with 10 fish per replicate treatment) approximately 5-8 mpf were collected from each treatment group (0, 0.3, 3, or 30 ppb atrazine), euthanized, and ovaries dissected. Zebrafish and gonads were weighed and GSI calculated. GSI among the atrazine treatment groups was not statistically different ($p=0.11$). Error bars are expressed as ± SD.

A

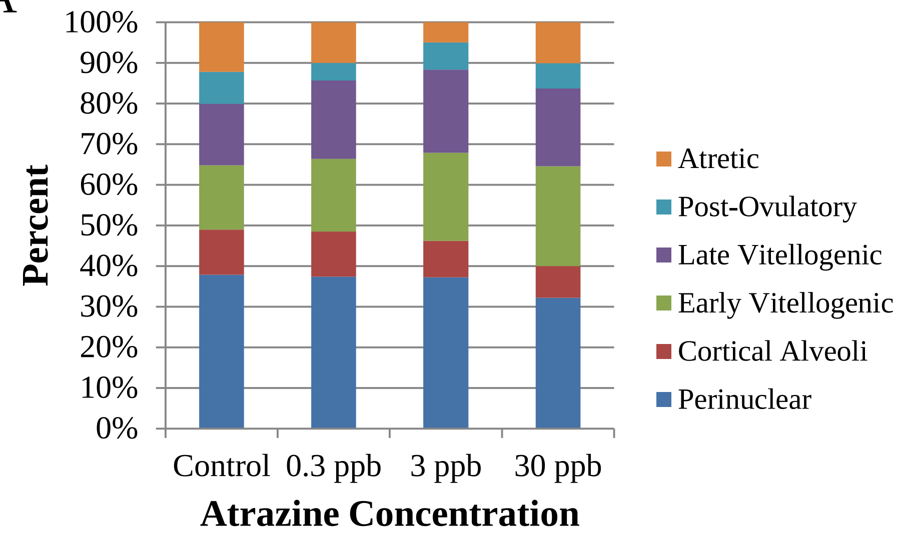

B

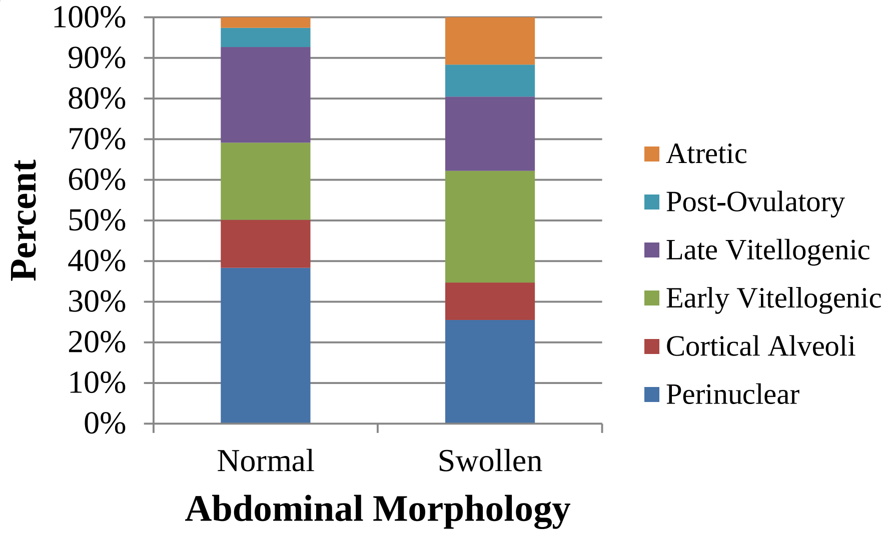

Supplementary Material, Figure S4. Follicular staging of adult female ovaries. Adult female zebrafish (4 replicates with 10 fish per replicate) approximately 5-8 mpf were collected from each treatment group (0, 0.3, 3, or 30 ppb atrazine), euthanized, and ovaries dissected and prepared for histological analysis. (A) The percent distribution of different oocyte stages across treatments was not significantly different from each other ($p>0.05$). (B) When comparing oocyte staging between females presenting with swollen abdomens which were unable to release eggs to normal females a significant difference was observed for the number of atretic follicles ($p=0.0002$).

Supplementary Material, Table S1. Genes altered in adult female gonad tissue in all three atrazine treatments.

Gene Symbol	Direction of Change	Log_2 Expression Ratio
A2M	Down	-1.300
ABCG4	Down	-0.621
ABHD12	Down	-0.961
ACBD6	Up	0.943
ACE	Up	1.400
ACHE	Up	1.549
ACTL6A	Down	-1.179
ADCY8	Up	0.853
ADCYAP1	Up	0.597
ADD3	Up	0.728
AGA	Up	0.645
AK1	Up	0.857
ALDH2	Down	-0.729
ALDH3A2	Up	0.947
Aldoart1	Up	0.639
ALKBH8	Up	1.288
AMPD3	Down	-1.010
ANLN	Down	-1.154
ANTXR2	Up	1.270
ANXA13	Up	0.656
AP1M2	Down	-1.052
APC2	Down	-0.825
APLP1	Up	0.976
AQP1	Up	0.976
ARFIP2	Down	-0.851
ARID3B	Down	-0.985
ARNTL2	Down	-1.083
ASIC2	Up	1.542
ATL3	Down	-2.054
ATP1A1	Up	0.822
ATP1B1	Down	-0.699
ATP6V0A2	Up	1.537
ATP6V0C	Down	-1.541
ATP6V1B2	Down	-0.656
ATPAF2	Up	0.863
BBS1	Down	-1.096
BBS12	Up	1.567
BCL11B	Down	-0.797
BET1	Up	0.948
BMS1	Down	-1.899

Gene Symbol	Direction of Change	Log_2 Expression Ratio
C11orf80	Down	-2.287
C12orf23	Down	-1.068
C17orf104	Down	-1.218
C1orf146	Down	-0.853
C1orf198	Up	0.769
C9orf114	Down	-0.632
C9orf172	Up	1.365
CA2	Up	0.959
CA8	Up	1.963
CADM1	Down	-1.366
CALB1	Down	-1.149
CAMK2N2	Up	0.813
CAMK4	Up	0.853
CAMKV	Up	0.791
CARTPT	Up	0.869
CASP8AP2	Down	-1.254
CBLN1	Up	1.170
CCDC69	Down	-0.900
CCNH	Down	-1.654
CDC27	Up	0.695
CDCP1	Down	-1.137
CDIP1	Down	-1.018
CENPM	Up	1.274
CEP70	Down	-1.146
CHAF1B	Down	-0.980
CIRBP	Up	0.626
CKM	Up	0.793
CLDN7	Down	-1.504
CLIP3	Up	0.736
CNN3	Down	-0.727
CPE	Up	0.939
CRHBP	Up	0.989
CSPG5	Up	0.973
CTDSP2	Up	1.097
CTNND2	Up	0.995
CTSF	Up	0.887
CYP19A1	Up	1.842
CYP1B1	Up	0.589
CYP27A1	Up	0.638
Dclk1	Down	-0.920
DDC	Down	-1.610
DDX19A	Up	0.664
DDX20	Down	-0.768

Gene Symbol	Direction of Change	Log_2 Expression Ratio
DDX4	Up	0.941
DEPDC1	Down	-0.817
DHX29	Down	-1.293
DNAJB4	Up	0.879
DNMT1	Down	-3.820
DPP7	Up	1.561
DPYSL3	Up	0.717
DRAM2	Up	2.180
DSPP	Up	1.287
DYNC1LI2	Down	-0.806
EFCAB1	Down	-0.594
EIF4G1	Down	-1.638
ELAVL3	Up	0.935
ELFN2	Up	1.477
ELOVL6	Down	-1.260
EN1	Down	-0.689
ENO4	Down	-1.873
ERI1	Down	-1.075
ETNK1	Up	0.819
EZH2	Up	0.875
FAM13B	Down	-0.970
FAM184B	Down	-0.622
FAM228B	Up	0.642
FAM69B	Down	-1.405
FAM83D	Down	-1.305
FAM84B	Down	-1.280
FANCF	Down	-1.332
FBXO15	Down	-1.084
FDXR	Down	-1.134
FKBP1B	Up	0.698
FLOT2	Up	0.702
Folh1	Down	-1.220
FOXA1	Up	0.746
FOXQ1	Down	-1.912
FOXR1	Up	1.011
GABRA6	Up	0.907
GAP43	Down	-1.300
GATA6	Up	0.588
GDF6	Up	0.927
GGCT	Down	-1.836
GLB1L2	Up	0.736
Gm19410	Up	0.749
GNB2	Down	-0.636

Gene Symbol	Direction of Change	Log_2 Expression Ratio
GNG13	Up	0.770
GNG3	Up	0.599
GP1BB	Down	-0.825
GPATCH8	Up	0.591
GPR157	Up	0.960
GPR85	Up	1.097
GPSM2	Up	1.057
GREB1L	Up	0.595
GRIA2	Up	0.850
GSAP	Down	-1.324
GSG1L	Up	0.718
GSR	Down	-1.217
GSS	Down	-0.828
GTSF1	Down	-0.973
H2AFV	Up	1.524
H2AFY2	Up	1.375
HBE1	Down	-0.735
HBZ	Up	1.065
HIF3A	Down	-1.078
HMBS	Down	-0.810
HPGD	Up	0.806
HTRA1	Up	1.031
IFFO1	Up	0.692
IGF2BP2	Down	-0.784
IGFBP1	Up	0.732
IGSF9B	Up	0.921
INSM1	Down	-0.909
Iqcd	Down	-0.754
IQCJ-SCHIP1	Up	0.674
ISLR2	Up	1.051
ISY1-RAB43	Down	-1.445
ITGB5	Up	0.833
KCNA1	Up	0.940
KCNA4	Up	0.653
KCNIP3	Down	-1.433
KIF1A	Down	-0.881
KIF20B	Down	-0.597
KIF23	Down	-1.118
KL	Down	-1.542
KLHDC10	Up	0.705
KMO	Down	-1.740
KPNA7	Up	0.739
KRT17	Up	0.829

Gene Symbol	Direction of Change	Log_2 Expression Ratio
L1CAM	Up	0.587
LDLRAP1	Down	-0.619
LEPREL4	Down	-0.652
LNX1	Up	1.349
LOC680121	Up	1.742
LRRC8D	Up	1.209
LRRN3	Up	0.616
LRRTM2	Up	0.655
MAB21L1	Up	1.220
MAF	Up	0.883
MAP2K5	Up	0.750
MAPK11	Up	0.759
MARVELD2	Down	-0.944
MBD3	Up	0.680
MC2R	Down	-1.095
MCOLN2	Up	0.666
MED6	Up	0.928
MEF2A	Down	-1.186
METTL20	Down	-0.690
METTL8	Up	0.647
MGAT5	Down	-1.195
MGST1	Up	0.699
MSI1	Up	0.694
MTA3	Down	-0.820
MTMR1	Down	-1.147
MTX3	Up	0.791
MYT1L	Up	1.233
NAF1	Down	-0.870
NAT8L	Up	2.412
NCAPH2	Up	1.275
NDRG2	Up	0.959
NDRG3	Up	0.975
NDRG4	Up	1.072
NDUFA4	Down	-0.671
NDUFS8	Down	-0.629
NEFL	Down	-0.588
NEGR1	Down	-1.442
NEK2	Down	-1.622
NELFCD	Up	0.746
NEUROD1	Up	0.716
NEUROD2	Down	-1.143
NIM1K	Down	-1.139
NMRK1	Up	1.163

Gene Symbol	Direction of Change	Log$_2$ Expression Ratio
NOVA1	Down	-0.862
NPC2	Down	-0.754
NPTX1	Down	-0.825
NRG2	Down	-1.586
Nrxn3	Down	-1.363
NSL1	Up	1.280
NUF2	Down	-1.028
ODF3	Up	1.975
OGG1	Up	0.644
Olfr146	Down	-0.674
OSBPL11	Down	-1.088
OSTF1	Down	-1.176
P2RX4	Down	-1.348
PANK1	Down	-0.813
PAQR4	Down	-0.732
PCDH19	Up	0.721
PCDH7	Down	-0.654
PCDHAC2	Down	-0.995
PCDHGA12	Up	2.398
PCLO	Up	0.814
PCMT1	Up	0.953
PCYT1A	Down	-1.064
PFAS	Down	-1.282
PHLDA2	Down	-0.742
PICK1	Down	-0.957
PIK3R1	Up	1.050
PIWIL1	Down	-0.710
PIWIL2	Up	0.745
PLP1	Down	-1.710
PLXNA4	Up	0.789
POLE	Up	1.052
POU3F3	Down	-1.568
Ppfia4	Up	1.040
PPP3R1	Up	0.762
PRIMPOL	Down	-0.928
PRTG	Up	0.811
PTBP1	Down	-0.844
PTGES	Up	1.036
PTPRD	Down	-0.893
PTPRS	Down	-1.002
PUSL1	Down	-0.696
PVALB	Down	-1.074
RAB13	Down	-0.977

Gene Symbol	Direction of Change	Log_2 Expression Ratio
RAD52	Down	-0.854
RAD54L	Down	-1.093
RALY	Up	0.861
RARA	Down	-0.656
RASD1	Up	1.254
RASL11B	Up	1.195
RASSF2	Up	2.522
RASSF8	Up	0.626
RBM24	Down	-1.566
RBM26	Down	-1.084
RBM41	Down	-1.696
RBMS2	Up	0.588
RCAN2	Up	0.774
RCAN3	Up	0.935
RCC2	Down	-0.710
RERGL	Up	0.855
RGL2	Down	-1.112
RGS20	Down	-0.663
RGS7BP	Down	-0.646
RIC8A	Up	0.620
RIMS2	Down	-0.776
RIPK4	Up	1.150
RND1	Up	2.020
RNF217	Down	-1.339
ROBO2	Up	0.723
RTEL1	Down	-0.771
RUNX1T1	Up	0.773
SCARB1	Up	1.240
SCG2	Up	0.618
SCN8A	Up	1.867
SDK1	Up	0.798
SEPT5	Up	1.854
SETD6	Up	0.773
SEZ6L2	Up	1.090
SFRP1	Down	-0.598
SGCE	Up	0.627
SH3D19	Down	-1.200
SHD	Up	1.646
SLC1A2	Down	-0.922
SLC1A4	Up	0.961
SLC25A19	Down	-1.448
Slc26a10	Down	-0.758
SLC27A1	Up	0.950

Gene Symbol	Direction of Change	Log_2 Expression Ratio
SLC35D1	Down	-0.639
SLC4A4	Up	0.699
SLC9A2	Down	-0.681
SMARCAL1	Down	-0.972
SNAI1	Down	-0.889
SNAP25	Up	1.482
SNX12	Up	0.927
SNX21	Down	-0.797
SORBS3	Down	-0.771
SOX1	Down	-1.473
SOX10	Down	-0.968
SPINT1	Down	-1.223
SPSB4	Up	1.053
SRGAP2	Up	0.702
ST6GAL1	Down	-1.162
STAR	Up	1.053
STARD8	Down	-1.105
STIL	Up	1.561
STMN2	Up	0.844
STOM	Down	-0.690
STX11	Up	1.813
SULT2B1	Up	1.155
SUN1	Up	1.065
SV2B	Down	-0.937
SVOPL	Up	1.156
SYCP1	Down	-2.075
SYNE1	Up	1.199
SYNGR3	Up	1.023
TAL1	Up	0.993
TAL2	Up	1.153
TBC1D15	Up	0.675
TDP2	Down	-0.921
TGDS	Down	-2.044
TIAL1	Down	-0.657
TJP2	Down	-0.714
TK1	Up	1.367
TMBIM1	Up	0.994
TMEM160	Down	-0.873
TMEM178B	Down	-0.964
TMEM35	Down	-0.699
TMEM51	Down	-0.889
TNFRSF19	Up	1.258
TONSL	Down	-0.715

Gene Symbol	Direction of Change	Log_2 Expression Ratio
Tor1aip1	Down	-0.885
TPPP3	Up	0.749
TRABD	Down	-1.068
TRAF2	Down	-0.720
TRAPPC2L	Up	0.838
TRIM16	Up	1.016
TRIM35	Down	-2.052
TRPM3	Down	-1.029
TSPAN15	Down	-1.186
TSPAN2	Up	2.381
TTLL7	Down	-1.380
TUBA1A	Up	0.792
TUBA3E	Up	0.855
TUSC3	Down	-1.010
UBE2C	Down	-0.714
UBE2J2	Up	0.883
UBE4A	Down	-1.280
UBR1	Down	-1.190
UHRF1	Up	1.051
UNC119	Down	-1.181
UNC119B	Up	1.169
URB2	Down	-0.923
USP44	Down	-0.925
VCAM1	Up	0.713
VGF	Up	0.950
VIM	Up	0.604
VIP	Down	-0.586
WBSCR27	Down	-0.784
WDR12	Down	-1.125
WDR26	Down	-0.810
WIF1	Up	0.634
XRCC2	Up	1.054
YAF2	Up	0.878
YRDC	Down	-0.863
YWHAG	Down	-1.230
ZC3H18	Down	-1.519
ZFPM2	Up	0.629
ZIC1	Down	-0.884
ZNF281	Down	-0.791
ZNF410	Up	2.856
ZNF598	Down	-0.817
ZNF91	Up	2.208

Supplementary Material, Table S2. Genes enrichment table in adult female gonad tissue at 0.3 ppb atrazine.

Physiological System Development and Function – 0.3 ppb	p-value[a]	Number of Genes[b]
**NERVOUS SYSTEM DEVELOPMENT AND FUNCTION**	2.88E-26 – 3.74E-4	565
**Morphology of CNS**	5.87E-18	135
**Neurotransmission**	1.96E-22	120
**Growth of neurites**	1.72E-15	117
**Myelination**	4.44E-6	38
**Coordination**	1.94E-9	54
**EMBRYONIC DEVELOPMENT**	2.51E-23 – 3.65E-4	396
**Development of head**	2.51E-23	203
**Development of body axis**	3.33E-21	213
**Growth of embryo**	8.24E-7	88
**BEHAVIOR**	5.00E-25 – 1.45E-4	260
**Behavior**	5.00E-25	228
**Cognition**	2.13E-14	101
**Learning**	3.96E-13	94
**Memory**	4.10E-6	48
**ORGANISMAL SURVIVAL**	3.97E-32 – 1.35E-4	497
**Organismal Death**	3.97E-32	491
**Perinatal Death**	5.99E-11	131
**Neonatal Death**	2.24E-9	98

[a] Derived from the likelihood of observing the degree of enrichment in a gene set of a given size by chance alone.

[b] Classified as being differentially expressed that relate to the specified function category; a gene may be present in more than one category.

Supplementary Material, Table S3. Gene enrichment table of adult female gonad tissue at 3 ppb atrazine.

Physiological System Development and Function – 3 ppb	p-value[a]	Number of Genes[b]
**NERVOUS SYSTEM DEVELOPMENT AND FUNCTION**	1.74E-15 – 1.62E-3	260
**Growth of neurites**	2.05E-10	57
**Migration of Neurons**	4.83E-6	27
**Morphology of CNS**	1.74E-15	73
**Dendritic Growth and Branching**	5.07E-8	27
**ORGAN MORPHOLOGY**	1.65E-13 – 1.62E-3	110
**Abnormal morphology of gonad**	6.52E-7	41
**Abnormal morphology of internal genitalia**	8.84E-6	37
**Mass of gonad**	4.01E-7	24
**Morphology of brain**	1.65E-13	65
**BEHAVIOR**	5.42E-18 – 1.56E-3	134
**Behavior**	5.42E-18	113
**Cognition**	1.69E-9	49
**Learning**	1.78E-8	45
**Memory**	8.91E-5	25
**ORGANISMAL SURVIVAL**	1.55E-18 – 3.03E-4	233
**Organismal Death**	1.55E-18	220
**Perinatal Death**	1.21E-5	56
**Neonatal Death**	1.25E-4	41

[a] Derived from the likelihood of observing the degree of enrichment in a gene set of a given size by chance alone.

[b] Classified as being differentially expressed that relate to the specified function category; a gene may be present in more than one category.

Supplementary Material, Table S4. Gene enrichment table of adult female gonad tissue at 30 ppb atrazine.

Physiological System Development and Function – 30 ppb	p-value[a]	Number of Genes[b]
**NERVOUS SYSTEM DEVELOPMENT AND FUNCTION**	7.61E-8 – 8.58E-3	184
**Morphology of CNS**	3.27E-7	48
**Synaptogenesis**	1.39E-3	15
**Growth of neurites**	1.67E-6	42
**Differentiation of neurons**	7.49E-5	32
**TISSUE DEVELOPMENT**	8.12E07 – 8.47E-3	152
**Gonadogenesis**	3.52E-03	32
**Development of genital organ**	3.40E-3	34
**Gametogenesis**	5.46E-03	26
**Development of neurons**	8.19E-04	16
**BEHAVIOR**	4.05E-10 – 6.51E-3	97
**Behavior**	4.05E-10	82
**Cognition**	3.30E-5	34
**Learning**	6.52E-4	29
**Locomotion**	4.24E-5	28
**ORGANISMAL SURVIVAL**	5.31E-10 – 5.71E-3	171
**Organismal Death**	5.31E-10	165
**Perinatal Death**	2.17E-4	45
**Neonatal Death**	5.85E-5	37

[a] Derived from the likelihood of observing the degree of enrichment in a gene set of a given size by chance alone.

[b] Classified as being differentially expressed that relate to the specified function category; a gene may be present in more than one category.

Supplementary Material, Table S5. Primers used in qPCR confirmation of microarray analysis of female gonad tissue.

Seq. ID*	Gene Symbol*	Primer Sequence
NM_131663.1	*STAR*	Forward: ATGCGACCAAGTGCAGATGA<br>Reverse: GCCATTCTGTCCCTGAGGTG
XM_005169447.1	*ACE*	Forward: AGAGGCATGGGGTCACAAAG<br>Reverse: CTGTGATTGCGAGATTGGCG
NM_131154.2	*CYP19A1*	Forward: CAGGGCATCATATTCAACTCAA<br>Reverse: AGGTGGTGCAGATCTCCATAGT
NM_001045256.1	*CRHBP*	Forward: GGATAACGAGATCAGCCCGG<br>Reverse: ACCCTCTACGGCCACCATAT
XM_689239.5	*HPGD*	Forward: AGCAAGGAGGGGCAATCATC<br>Reverse: ATGGCTCGTGAAAACCCGAT
NM_001045256.1	*CYP1B1*	Forward: CAACGGTTACCCCATTCCCA<br>Reverse: GCCGAGTGAGAAGATGAGCA
NM_001114553.2	*VIP*	Forward: TACCCTTCGCCTCCATGAGA<br>Reverse: TCATCACTGACCCGCTTTCC
NM_181601	*B-ACTIN*	Forward: CTAAAAACTGGAACGGTGAAGG<br>Reverse: AGGCAAATAAGTTTCGGAACAA

*Seq IDs are zebrafish and gene symbols are the human homolog.
